# Supplementary material for: No tension between assembly models of super massive black hole binaries and pulsar observations
Source: Nat Commun. 2018 Feb 8;9:573. doi: 10.1038/s41467-018-02916-7 (PMC5805789; doi:10.1038/s41467-018-02916-7)
Supplement: Supplementary file 1 — Supplementary Information [file 41467_2018_2916_MOESM1_ESM.pdf]

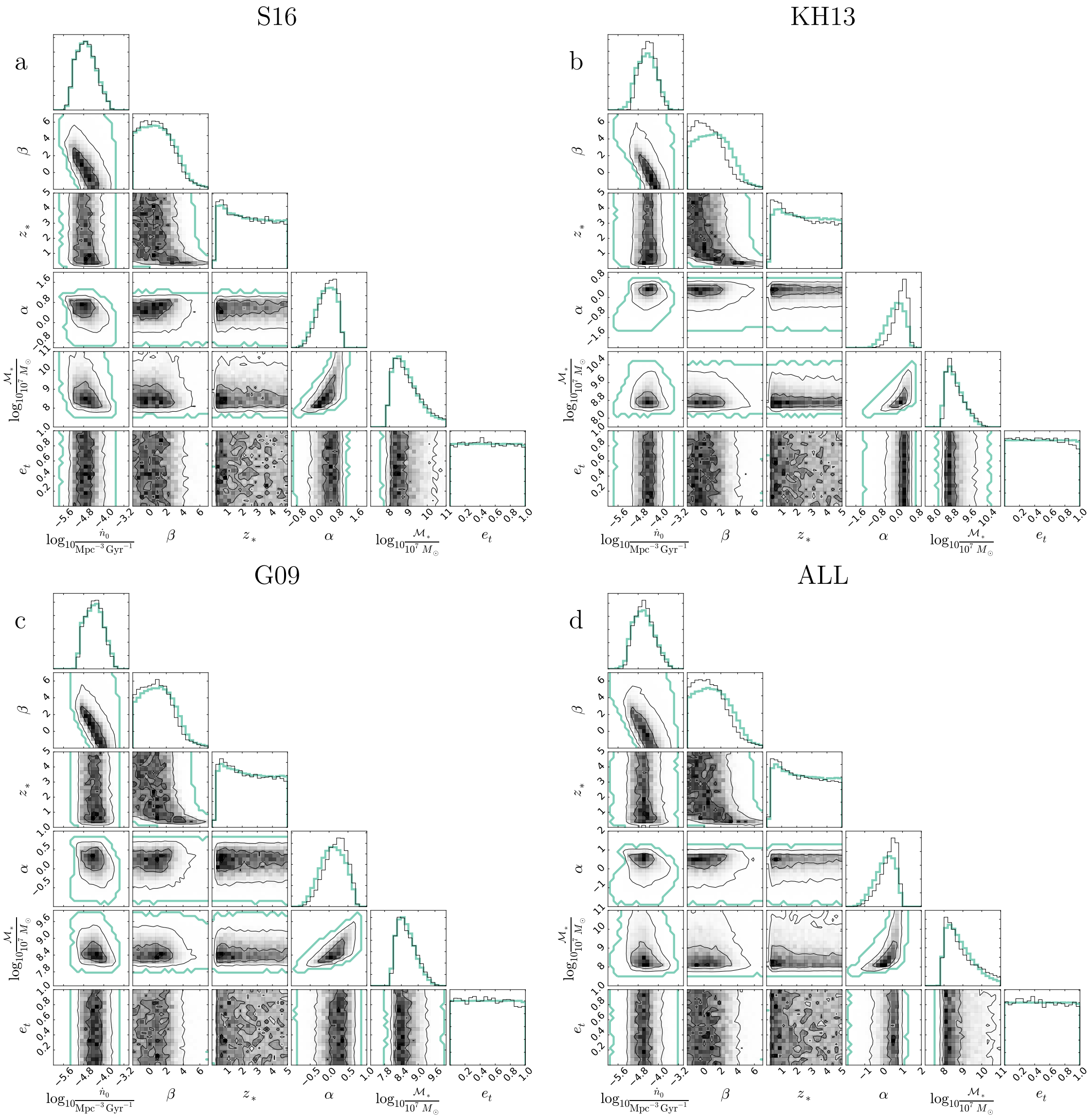

Supplementary Figure 1: **Posterior density function on the model parameters given the PPTA upper limit.** The nested sampling algorithm (see Methods in the main text) returns the full posteriors of the N-dimensional parameter space and the model evidences (see Supplementary Table 2). Here we show the posteriors for our main analysis of the PPTA upper limit using the default six parameter model ($\theta = \dot{n}_0, \beta, z_*, \alpha, \mathcal{M}_*, e_{\mathrm{t}}$). The four panels show the result for each of the astrophysical model priors: (a) top left S16; (b) top right KH13; (c) bottom left G09; (d) bottom right ALL. Within each panel, the plots on the diagonal show the one-dimensional marginalised distributions for each parameter with the posterior (thin-black) and prior (thick-green). The central plots show the two-dimensional posterior distributions for each parameter pair (grey-shading) along with the extent of the prior (thick-green contour). By comparison of the prior and posterior we see that the current PTA observations impose little constraint on the shape of the SMBHB mass function. For S16, the most conservative model (top left), the prior and posterior are virtually identical. Even for the KH13 model (top right), the two distributions match closely, with only appreciable differences for β and α due to the PPTA limit excluding the highest values of h_{c} predicted by the model (cf main text Fig. 1) resulting in a preference for large α and negative β. In fact, for the mass function adopted in equation 2 of the main text, a large α results in a SMBHB population dominated by low mass systems, which tends to suppress the signal. Likewise, a small (or negative) β implies a sparser population of SMBHB at higher redshift, again reducing the GW background level. In any case, current PTA measurements provide little new information on the SMBHB cosmic population as also demonstrated by the small K-L divergences between prior and posterior of individual model parameters in Supplementary Table 1.

Supplementary Table 1: Kullback-Leibler divergences for individual model parameters given the PPTA upper limit. The K-L divergences in the table compare the marginalised prior and posterior distributions for the individual parameters in the default 6-parameter model ($\theta = \dot{n}_0, \beta, z_*, \alpha, \mathcal{M}_*, e_\mathrm{t}$) and given the PPTA upper limit. The K-L divergence is a measure of the degree of similarity between two probability distributions with zero being identical. The rows indicate the four astrophysical models used as priors in this study (KH13, G09, S16, ALL). As also seen qualitatively from Supplementary Fig. 1, there is little difference between the prior and posterior for all four models. KH13, the model predicting the highest characteristic strain as expected has the highest K-L divergences, however as also shown by Supplementary Fig. 1 the difference is still small.

Model	K-L divergence					
	$\log_{10}\dot{n}_0$	β	z_*	α	$\log_{10}\mathcal{M}_*$	e_t
KH13	0.06	0.05	< 0.01	0.24	0.03	< 0.01
G09	< 0.01	0.01	< 0.01	0.04	0.01	< 0.01
S16	< 0.01	< 0.01	< 0.01	0.01	< 0.01	< 0.01
ALL	0.02	0.02	< 0.01	0.08	0.02	< 0.01

Supplementary Table 2: Progression of model constraints with improving upper limits. The table shows the natural logarithm of model evidences and associated h_c Kullback-Leibler (K-L) divergences (in parenthesis) for each of the astrophysical models: KH13, G09, S16 and ALL. We consider two different parametrisations of the SMBHB dynamics; one which has only e_t as a free parameter (column 'e_t', the 6 parameter model), and one where we add the normalization factor η to the density at the influence radius ρ_i as a free parameter (column '$e_t + \eta$', the 7 parameter model). Numbers are reported for three values of the 95% PTA upper limit; the current upper limit at 1×10^{-15} and two possible future upper limits at 3×10^{-16} and 1×10^{-16}. See also Supplementary Note 1.

Model	$h_{1yr,95\%} = 1 \times 10^{-15}$		$h_{1yr,95\%} = 3 \times 10^{-16}$		$h_{1yr,95\%} = 1 \times 10^{-16}$	
	e_t	$e_t + \eta$	e_t	$e_t + \eta$	e_t	$e_t + \eta$
KH13	−2.36 (0.85)	−2.23 (0.84)	−5.68 (2.25)	−5.47 (2.25)	−13.17 (5.18)	−9.03 (7.11)
G09	−1.2 (0.39)	−1.1 (0.39)	−3.35 (1.11)	−3.17 (1.09)	−8.26 (2.86)	−6.38 (4.02)
S16	−0.6 (0.37)	−0.57 (0.38)	−1.62 (0.69)	−1.6 (0.71)	−3.82 (1.42)	−3.56 (1.48)
ALL	−1.23 (0.62)	−1.14 (0.62)	−2.68 (1.33)	−2.63 (1.31)	−5.74 (2.50)	−5.09 (2.53)

Supplementary Note 1
The progression of constraints given improvements on the upper limit.

From Supplementary Table 2 we see that, given the current upper limit of $h_{1\mathrm{yr},95\%} = 1 \times 10^{-15}$, there are no significant differences between the 6 and 7 parameter results, with virtually identical evidences and K-L divergences. Together with the flat e_t posteriors shown in Supplementary Fig. 1, this leads us to an important conclusion: current PTA non detections do not favour (nor require) a strong coupling with the environment. Neither high stellar densities (i.e. efficient 3-body scattering) nor high eccentricities are preferred by the data. The conservative S16 model is always favoured, however even when compared to KH13, one obtains $\ln \mathcal{B} = 1.76$, which only mildly favours S16 [1]. In addition, all K-L divergences are smaller than unity, indicating only minor updates with respect to the h_c prior distributions. This is another measure of the fact that the data are not very informative.

Turning to the limit at $h_{1\mathrm{yr},95\%} = 3 \times 10^{-16}$, the K-L divergences of all models, with the exception of S16, are now larger than unity indicating that the upper limit is becoming more informative. In terms of model comparison, S16 is now mildly favoured with respect to G09 ($\ln \mathcal{B} = 1.73$) and strongly favoured compared to KH13 ($\ln \mathcal{B} = 4.06$). Again, we note that adding η does not make a significant difference to the model evidence. Even with such a low upper limit, neither high eccentricity nor strong coupling with the environment improve the agreement between model expectations and data. Although this seems counter-intuitive, we should keep in mind that the upper limit is set around $f \approx 5 \times 10^{-9}$ Hz (cf main text Fig. 1). Any dynamical effect should therefore cause a turnover of the spectrum around 10^{-8} Hz to have an impact on model selection, which occurs only in a small corner of parameter space where both e_t and η are high. However, for all models $h_{1\mathrm{yr},95\%} = 3 \times 10^{-16}$ is still consistent with the tail of the h_c distribution when an $f^{-2/3}$ spectrum is assumed. Invoking high e_t and η is not necessary.

The limit becomes far more interesting if it reaches $h_{1\mathrm{yr},95\%} = 1 \times 10^{-16}$. Now all K-L divergences are substantial, indicating that the measurement is indeed informative. Model selection now strongly favours model S16 compared to any other model, whether η is included or not. Even including all environmental effects, we find that S16 is decisively preferred over KH13 with $\ln \mathcal{B} = 5.47$. Note however, that S16 has a log evidence of -3.56 of its own, considerably lower than zero (the evidence of a model is unaffected by the measurement). Since delays and stalling can potentially decrease the GW background by preventing many SMBHB from merging, it is likely that a non detection at this level will provide strong support for those dynamical effects. These are not yet included in our modelling and we plan to explore them in the future.

Supplementary Table 3: SMBH-galaxy relations used to construct the astrophysical models. For each merging galaxy pair, we assign a black holes with masses drawn from 14 SMBH-galaxy relations found in the literature. In the table we list the relations used. The total black hole mass is described by the relation $\log_{10} M = a + b \log_{10} X$. Here $X = \{\sigma/200 \text{ km s}^{-1}, L_{\rm i}/10^{11} \text{L}_\odot \text{ or } M_*/10^{11} \text{ M}_\odot\}$, with σ being the stellar velocity dispersion of the galaxy bulge, $L_{\rm i}$ its mid-infrared luminosity, and M_* its bulge stellar mass. Each relation is also characterized by an intrinsic scatter ϵ. In the table, we list the parameters a, b and ϵ for each of the relations taken from the literature. There are two entries for Graham (2012) [2] who proposes a double power law with a break at $\bar{M}_* = 7 \times 10 \text{ M}_\odot$, for which the values in parenthesis refer to $M_* < \bar{M}_*$.

Paper	X	a	b	ϵ
Häring & Rix (2004) [3]	M_*	8.2	1.12	0.30
Sani et al. (2011) [4]	M_*	8.2	0.79	0.37
Beifiori et al. (2012) [5]	M_*	7.84	0.91	0.46
McConnell & Ma (2013) [6]	M_*	8.46	1.05	0.34
Graham (2012) [2]	M_*	8.56	1.01	0.44
		(8.69)	(1.98)	(0.57)
Kormendy & Ho (2013) [7]	M_*	8.69	1.17	0.29
Sani et al. (2011) [4]	$L_{\rm i}$	8.19	0.93	0.38
Gültekin et al. (2009) [8]	σ	8.23	3.96	0.31
Graham et al. (2011) [9]	σ	8.13	5.13	0.32
Beifiori et al. (2012) [5]	σ	7.99	4.42	0.33
McConnell & Ma (2013) [6]	σ	8.33	5.57	0.40
Graham & Scott (2012) [10]	σ	8.28	6.01	0.41
Kormendy & Ho (2013) [7]	σ	8.5	4.42	0.28
Shankar et al. (2916) [11]	σ	7.8	4.3	0.3

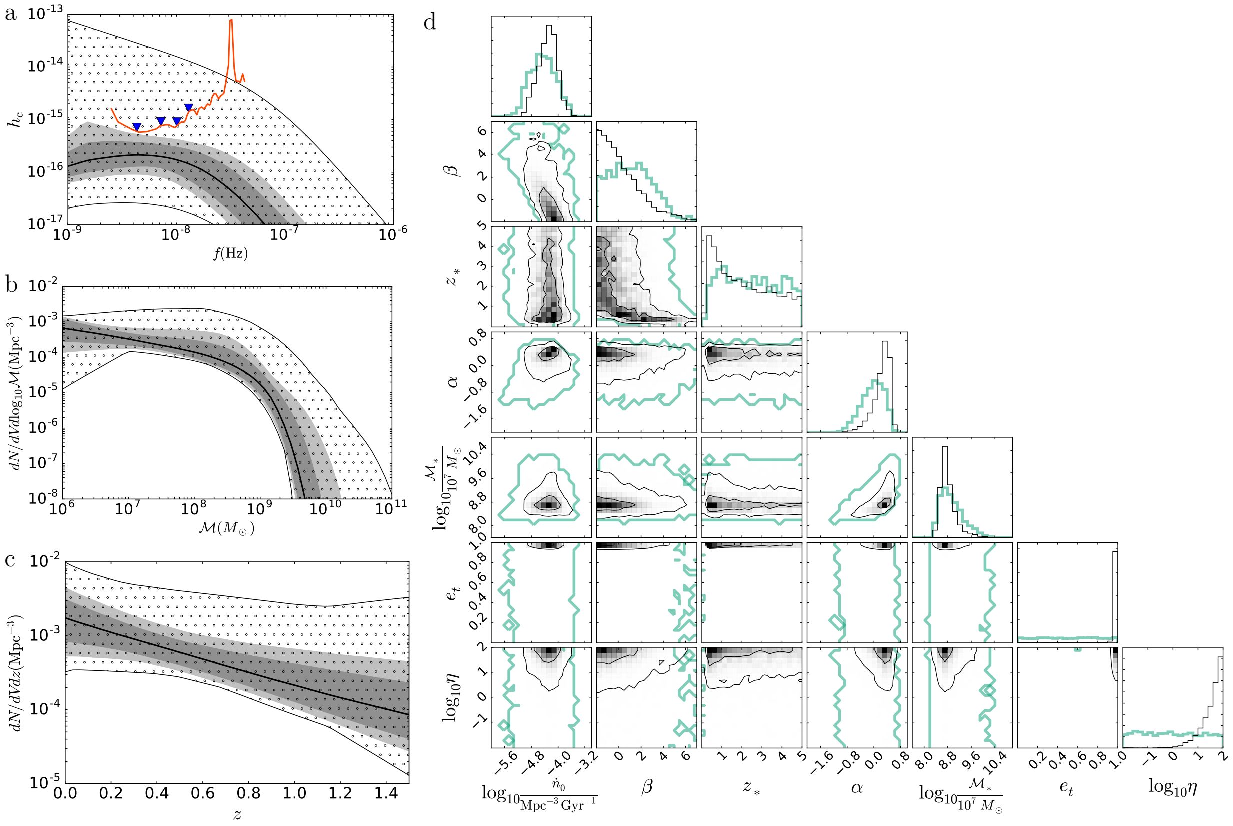

Supplementary Figure 2: **Posterior distrubtions for a putative upper limit at $h_{1\mathrm{yr}} = 1 \times 10^{-16}$.** Here we show results of a upper limit of $h_{1\mathrm{yr},95\%} = 1 \times 10^{-16}$ for the six parameter model and astrophysical prior KH13. Left panel, from top to bottom: (a) characteristic amplitude, (b) density mass function and (c) density redshift evolution of SMBHBs. In each panel, the dotted areas represent the astrophysical prior, the shaded bands are the 68% and 90% of the posterior distribution and the solid thick line is its median value. In the top panel only, the solid orange curve represents the bin-by-bin 95% upper limits at different frequency bins (with blue triangles indicating the frequency bins we use), resulting in an overall limit $h_{1\mathrm{yr},95\%} = 10^{-16}$. Right panel: (d) the individual posterior distributions. The diagonal plots show the one-dimensional posterior distribution (black) along with the prior (green-thick), whilst the central plots show the two-dimensional posterior for each of the parameter pairs again with the extent of the prior shown by the single green-thick contour. We see that now all the posteriors differ significantly from the respective prior. Low β and z_* are preferred, because this suppresses the total number of SMBHs at high redshifts. Note that higher values of $\dot{n}_0$ are preferred. Although this might be surprising, it is dictated by the shape of the prior of dn/dz (lower left panel); in order to minimize the signal, it is more convenient to allow a negative β at the expenses of a higher local normalization $\dot{n}_0$ of the merger rate. High α values are clearly preferred, since they imply a population dominated by low mass SMBHBs (middle left panel). The e_{t} posterior now shows a prominent peak close to the maximum $e_{\mathrm{t}} = 0.999$, with a long tail extending to zero (right panel, final plot). Very high eccentricities are preferred, although low values are still possible. This is because 1×10^{-16} is only a 95% upper limit, therefore there is a small chance that a low eccentricity model producing a signal surpassing the 1×10^{-16} value is nonetheless accepted in the posterior.

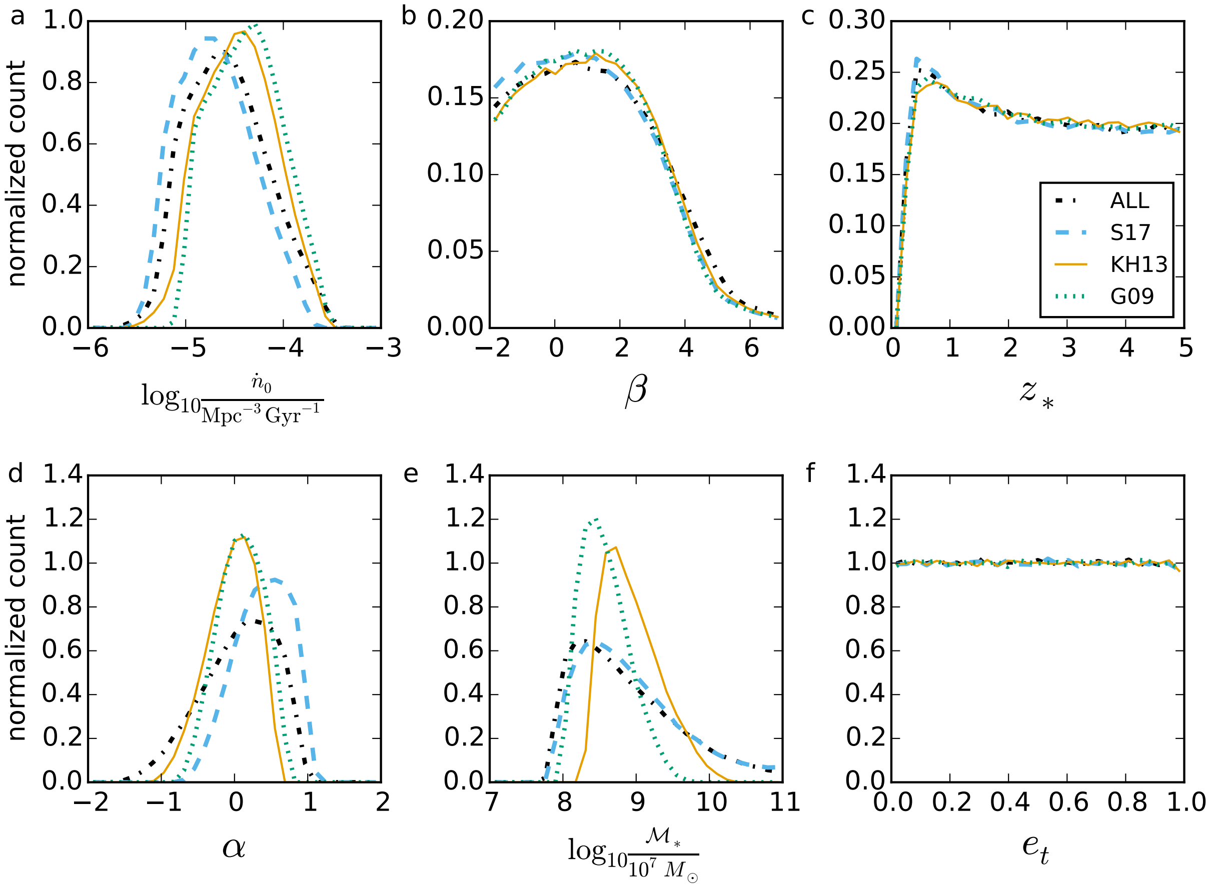

Supplementary Figure 3: **Prior density functions of the model parameters.** The numerical SMBHB mass functions obtained from the prior bands in main text Fig. 3 need to be related analytically to the parameters of expression 2. Our strategy is therefore to make a large series of random draws of the five parameters defining equation 2, and to retain only those sets that produce dn/dz and $dn/d\mathcal{M}$ within the boundaries set by the empirical models shown in Fig. 3 of the main text. The prior distributions for the individual parameters obtained in this way are shown here. Top row from left to right: (a) $\dot{n}_0$; (b) β; (c) z_*; bottom row from left to right: (d) α, (e) $\mathcal{M}_*$, (f) $e_{\rm t}$. The lines represent the priors for the four astrophysical models KH13 (orange, solid), S16 (blue, dashed), G09 (green dotted) and ALL (black dash-dot). We see that the redshift parameters (β and z_*) have a very similar prior for each of the models. The main differences are seen in the number rate density of mergers $\dot{n}_0$ and in the mass distribution parameters (α and $\mathcal{M}_*$). KH13 and ALL prefer higher values of $\dot{n}_0$. S16 allows for slightly higher values of α (in comparison to KH13 and G09), corresponding to a more negative slope on the mass distribution, with preference for a larger number of low mass binaries. The eccentricity parameter $e_{\rm t}$ is unaffected by the choice of model, therefore we place on it a flat prior in the range $10^{-6} < e_{\rm t} < 0.999$.

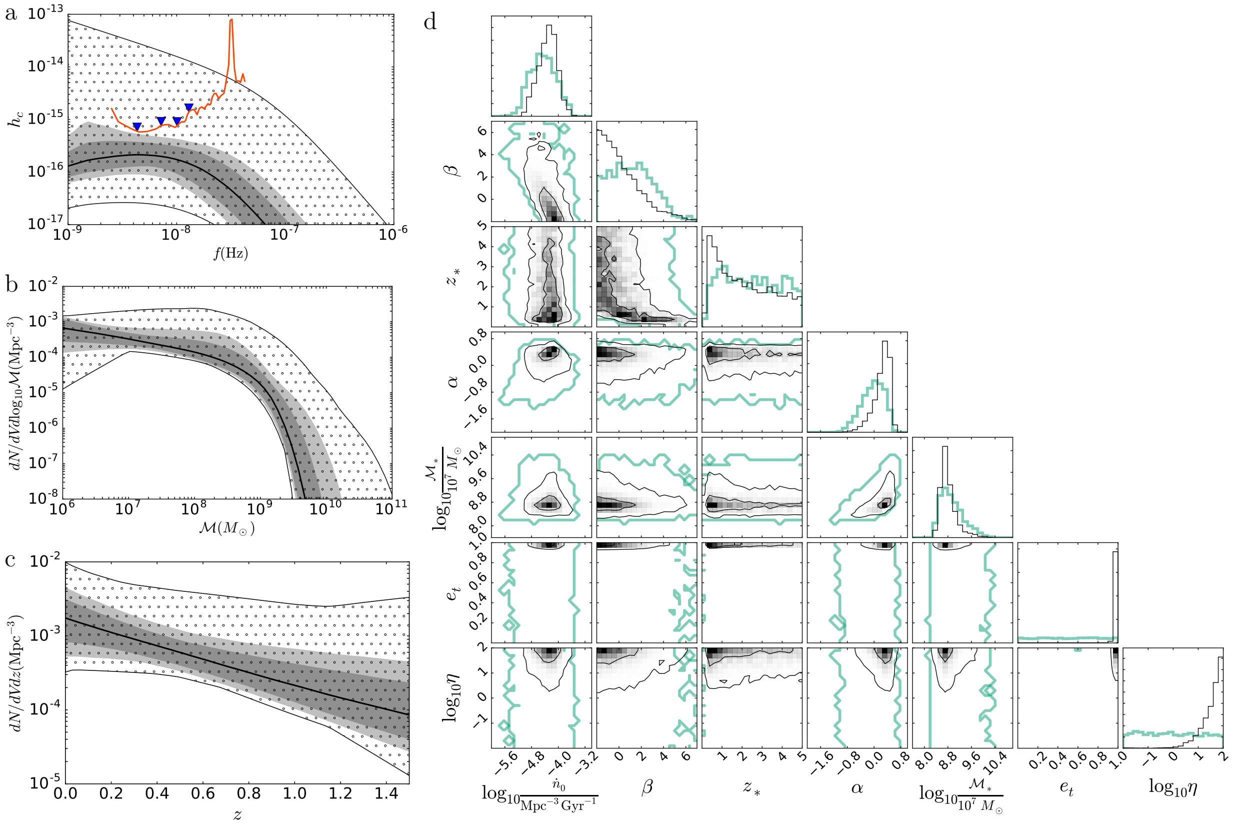

Supplementary Figure 4: Posterior distrubtions for a putative upper limit at $h_{1\mathrm{yr}} = 1 \times 10^{-16}$ with the addition of a free stellar density parameter. Here we show results of a upper limit of $h_{1\mathrm{yr},95\%} = 1 \times 10^{-16}$ for the seven parameter model and astrophysical prior KH13. The plots shown are identical to the description of Supplementary Fig. 2, the only difference being the addition of the stellar density parameter η in our analysis. Most notably, we see now that extremely high eccentricities and high densities are strongly favoured in the right panel (d). This is primarily because the addition of η extends the prior in h_c (shown in (a), the upper left panel) downwards quite below the level imposed by the upper limit. It is therefore now easier to find points in the parameter space consistent with the measurement when e_t and η are large. Should other SMBH-host galaxy relations being ruled out by independent constraints, a PTA upper limit of 1×10^{-16} would provide strong evidence of surprisingly extreme dynamical conditions of SMBHBs.

Supplementary References

[1] R. E. Kass and A. E. Raftery. Bayes factors. *Journal of the American Statistical Association*, 90:773–795, 1995.

[2] A. W. Graham. Breaking the Law: The M $_{bh}$-M $_{spheroid}$ Relations for Core-Sérsic and Sérsic Galaxies. *Astrophys. J.*, 746:113, February 2012.

[3] N. Häring and H.-W. Rix. On the Black Hole Mass-Bulge Mass Relation. *Astrophys. J. Letters*, 604:L89–L92, April 2004.

[4] E. Sani, A. Marconi, L. K. Hunt, and G. Risaliti. The Spitzer/IRAC view of black hole-bulge scaling relations. *Mon. Not. R. Astron. Soc.*, 413:1479–1494, May 2011.

[5] A. Beifiori, S. Courteau, E. M. Corsini, and Y. Zhu. On the correlations between galaxy properties and supermassive black hole mass. *Mon. Not. R. Astron. Soc.*, 419:2497–2528, January 2012.

[6] N. J. McConnell and C.-P. Ma. Revisiting the Scaling Relations of Black Hole Masses and Host Galaxy Properties. *Astrophysical Journal*, 764:184, February 2013.

[7] J. Kormendy and L. C. Ho. Coevolution (Or Not) of Supermassive Black Holes and Host Galaxies. *ARA&A*, 51:511–653, August 2013.

[8] K. Gültekin and et al. The M-σ and M-L Relations in Galactic Bulges, and Determinations of Their Intrinsic Scatter. *Astrophysical Journal*, 698:198–221, June 2009.

[9] A. W. Graham, C. A. Onken, E. Athanassoula, and F. Combes. An expanded M_{bh}-σ diagram, and a new calibration of active galactic nuclei masses. *Mon. Not. R. Astron. Soc.*, 412:2211–2228, April 2011.

[10] A. W. Graham and N. Scott. The (black hole mass)-(host spheroid luminosity) relation at high and low masses, the quadratic growth of black holes, and intermediate-mass black hole candidates. *ArXiv e-prints*, November 2012.

[11] F. Shankar and et al. Selection bias in dynamically measured supermassive black hole samples: its consequences and the quest for the most fundamental relation. *MNRAS*, 460:3119–3142, August 2016.
